# Adolescents’ exposure to secondhand smoke and its association with susceptibility to smoking and mental health in Lagos, Nigeria

**DOI:** 10.11604/pamj.2023.44.202.35973

**Published:** 2023-04-26

**Authors:** Afolabi Oyapero, Olufemi Erinoso, Olubukola Olamide Olatosi

**Affiliations:** 1 Department of Preventive Dentistry, Lagos State University College of Medicine, Lagos, Nigeria,; 2 Department of Oral and Maxillofacial Surgery, Lagos State University Teaching Hospital, Lagos, Nigeria,; 3 Department of Child Dental Health, Faculty of Dental Sciences, College of Medicine University of Lagos, Lagos, Nigeria

**Keywords:** Anxiety, depression, secondhand smoke, smoking susceptibility, tobacco

## Abstract

**Introduction:**

this study assessed the relationship between exposure to secondhand smoking (SHS) and its association with self-reported anxiety, depression and susceptibility to smoking among adolescents in Lagos, Nigeria.

**Methods:**

depression among study subjects was determined using the Patient Health Questionnaire-9 (PHQ-9) while the Generalized Anxiety Disorder - 7 (GAD-7) was used to determine anxiety levels. Susceptibility to smoking cigarettes was also determined while the Statistical Package for the Social Science (SPSS) 26.0 software was used for data analysis. Significant associations were determined at P-values <0.05.

**Results:**

of the 300 adolescents surveyed (mean age 12.9±1.43), 7.6 % were regularly exposed to SHS, of which 3.0% were daily exposed to SHS indoors. In multivariable analyses, indoor SHS exposure for ≥ 1 hour daily was associated with increased odds for susceptibility to smoking (AOR=3.793; 95%-CI: 0.98-14.60; p= 0.052) and increased odds for depression (AOR=1.303; 95%-CI: 0.84-2.01; p= 0.228) and slightly reduced odds for anxiety (AOR=0.952; 95%-CI: 0.62-1.47; p=0.822).

**Conclusion:**

secondhand smoking exposure was associated with higher odds of susceptibility to smoking cigarettes and depression among adolescents exposed to SHS, especially among females living in cramped accommodations. Further validation of these findings should however be determined by cohort study designs.

## Introduction

Secondhand smoke (SHS) is the inadvertent inhaling of tobacco smoke by those who are not active smokers. It consists of the mainstream smoke inhaled by a smoker [1] and side stream smoke with a higher concentration of toxins and carcinogens [2]. The noxious chemicals in SHS include polycyclic aromatic hydrocarbons, nitrosamines, tar and carbon monoxide, carbonyls [3]. The harmful effects of SHS on adults, children, pregnant women and their fetuses has been extensively documented by many researchers with sufficient evidence to infer a causal relationship [4-6]. These harmful effects of exposure to SHS [7] include fetal DNA damage, low birth weight, childhood respiratory infections, asthma, lower levels of breast-feeding, poorer academic performance, lung cancer, coronary heart disease and malignancies [8-10]. This range of health consequences can also significantly impact adolescents. Adolescence is a period of significant physical, psychological and social transitions as well as vulnerability to environmental stresses. During this period, the adolescent develops enhanced cognitive functions in areas such as decision-making and impulse control. In addition to tobacco smoke, SHS is one of the potential hazards that could potentially impact on adolescents´ health and well-being. Global reports from tobacco monitoring agencies have documented that 10% of adolescents are exposed to SHS in residential areas and in the public [11]. Similarly, a nationwide survey conducted among adolescents in US in 2017 reported an annual increase in SHS, with over 55% of them being exposed [12]. Thus, the potential adverse effect of SHS on their physical and psychological well-being necessitates adequate investigation. The negative impact of SHS on mental health is postulated to occur through the dopaminergic systems and the initiation of chronic stress [13,14]. Some studies have demonstrated the effects of SHS on the incidence of depressive symptoms [15-18]. While some reported significantly positive associations, [15,16] others observed weak associations [17,18]. Recently, a study of Chinese adolescents documented that SHS exposure for more than 5 days a week doubled the risk of depression among them [19]. These findings are important because depression in childhood predisposes to recurrent depression, alcoholism and suicidal tendencies [20], as well as somatic illnesses [21]. Furthermore, depressive disorders in adolescence is strongly correlated with depression in adulthood [20]. There is also a possibility that adolescents exposed to SHS could initiate the smoking habit since most smokers started the habit during adolescence and became more prone to heavy smoking in adulthood, often finding it difficult to quit [22]. Previous metanalysis have established the association between exposure to secondhand smoking and depressive symptoms among in-school adolescents and also among pregnant women [23,24].

Susceptibility to smoking, a tool developed by Pierce *et al*. [25] has been validated for identifying adolescents who are likely to initiate the smoking habit. Exposure to SHS has been identified as a risk factor that can increase susceptibility to cigarette smoking as well as nicotine dependence [26]. The importance of susceptibility to smoking within the developmental context of adolescence is very noteworthy, because of the role of behavioral intentions in predicting future behavior [27]. Researchers have previously documented that a very high proportion of adolescents who smoke live in low- and middle-income countries (LMICs) [28]. This is troubling, since the initiation of tobacco smoking during early adolescence is strongly correlated with persistent smoking during adulthood [29]. Inadequate attention has been given to the exposure of adolescents to SHS in Nigeria and its effect on their mental health and susceptibility to uptake of the smoking habit [30]. Studies among adolescents are crucial, because the developing brain of adolescents is particularly susceptible to substances found in SHS [31]. Secondly, data from LMICs are scarce. This is omission is not acceptable because the enforcement of tobacco control policy legislation is very weak in LMICs, compared with high-income countries [32]. This study thus aimed to determine the relationship between exposure to SHS and self-reported anxiety, depression and susceptibility to smoking among adolescents in Lagos, Nigeria.

## Methods

**Study design and settings:** this descriptive study was conducted between April and July 2021, in Lagos State, Nigeria. The population of Lagos State is about 20 million people, who are widely distributed in several rural and urban communities. It is a densely populated State and almost half of the commercial activities in the country take place in the city. Lagos State has one of the largest private basic education services globally and private schools provide services for almost 60% of the students in the State.

**Study participants and sampling method:** data were collected using randomly selected sample of Nigerian adolescents aged between 12 to 17 years. A multi-stage random sampling method was utilized to enlist the study participants. A simple random technique by balloting method was used to select 2 LGAs (Ikorodu and Ikeja) in the first stage. At the second stage, a sampling frame of secondary educational institutions was obtained from the Lagos State Ministry of Education website and two schools in each LGA were randomly selected by balloting. The third stage involved selection of eligible classes in the schools by simple random sampling. Children who met the eligibility criteria for the study were enlisted proportionally in each school using a table of random numbers with the class nominal roll serving as a sampling frame.

**Sample size calculation:** the minimum sample size was obtained using the formula for cross-sectional studies below:


$$n=\frac{Z^{2}pq}{d^{2}}$$

Where: n= minimum sample size for the study; Z= 1.96 at 95% confidence level and p= proportion of adolescents who were exposed to SHS; q= 1-p; and d= acceptable margin of error of 5% precision. Using a prevalence of 15.5% for household exposure to SHS in a previous study [33]. n= (1.96)^2^ x 0.15 x 0.85= 3.841 x 0.15 x 0.85 =196. Provision for incomplete responses of 20% gave a final sample size of 274 respondents.


$$n={\left(1.96\right)}^{2}\times 0.15\times 0.85=3.841\times 0.15\times 0.85=196$$

**Inclusion criteria:** these included students attending junior and senior secondary schools aged between 12 -17 years, with no history of tobacco use; those who gave their assent (for pupils aged 12 years), gave their consent (for students 13 years and older) and had parental consent (for all students) were selected for the study.

**Exclusion criteria:** students who were less than 12 years or more than 17 years of age; those that withheld their assent or consent and those without parental consent; physically, mentally or medically challenged pupils, as well as those who had any history of personal tobacco use, were also excluded from the study.

**Data collection tool:** a validated interviewer administered questionnaire which was partly modified from the Global Youth Tobacco Survey (GYTS) version 1.2 2014 was utilised. It obtained sociodemographic information, history of exposure to SHS, assessment of depression with the Patient Health Questionnaire-9 (PHQ-9), assessment of anxiety with the Generalized Anxiety Disorder - 7 (GAD-7) and susceptibility to smoking cigarettes. Section A); determined the socio-demographic characteristics of the participants. Section B); comprised questions about the smoking behaviour of parents and household members as well as the child´s exposure to SHS. The independent variable was SHS exposure, and this was assessed with the following questions: a) ´Within the previous seven days, did someone smoke cigarettes indoors in your home with you present?´ and b) ´Within the previous seven days, did anyone smoke in your presence at any other location apart from your home?´. Those who responded ‘0 days´ were categorized as unexposed to SHS, while those who responded: ´1-2 days´, ´3-4 days´, ´5-6 days´ and ´all seven days´ were categorized as exposed to SHS. In Section C, the 7-item GAD-7 scale, was used to assess self-reported symptoms and severity of anxiety respectively. The total scores of GAD-7 were categorized as normal (0-5), mild (6-10), moderate to severe (≥11) anxiety. The PHQ-9, a 9-item self-report questionnaire was used to assesses depression symptoms and severity in Section D. The PHQ-9 questionnaire was scored from 0 to 27, with higher scores indicating more severe depressive symptoms. The total score was interpreted as indicating none-minimal depression (score 0-4), mild depression (score 5-9), moderate depression (score 10-14), or moderately severe depression (score 15-19), and severe depression (score 20-27). In Section E; susceptibility to smoking cigarettes, indicated by the lack of a strong choice to abstain from smoking, was assessed using a composite index of three questions: a) if a friends gave you a cigarette, would you smoke it? b) are you likely to smoke a cigarette at any time within the next year? and c) How likely is it that you will be smoking cigarettes by the time you are 18 years old? Any response other definitely not to any of the three items indicated that the respondent is susceptible.

**Statistical analysis:** statistical analysis was conducted with SPSS version 26.0 software (Version 26.0. Armonk, NY: IBM Corp). Descriptive statistics were presented as frequencies and percentages while bivariate associations were analyzed using chi-squared and Anova tests. Bivariate and multivariate logistic regression analysis was done using sociodemographic variables and exposure to SHS as the predictor variables to identify outcome variables of interest which were self-reported anxiety, depression and susceptibility to smoking. Statistical significance was determined at p values <0.05.

**Ethical aspects:** ethical approval was obtained from the Health Research and Ethics Committee of the Lagos State University Teaching Hospital (Approval number: LREC/06/10/1798) and informed assent and consent was obtained from participants.

## Results

Characteristics of participants are summarized in Table 1. Of the 300 adolescents surveyed (mean age 12.9±1.43), 66 (22%) were exposed to SHS of which 34 (11.4%) were rarely exposed, 23 (7.6%) were often exposed while 9 (3.0%) of the children were daily exposed to SHS indoors. In the bivariate analysis, females (p=0.022), those living in cramped accommodations (p=0.020), and those <5 years of age when their caregivers started smoking indoors (p=0.001) had significantly higher odds of exposure to SHS (Table 1). Table 2 shows the bivariate association between secondhand smoke exposure, anxiety, depression and susceptibility to smoking among the study participants. The prevalence of self-reported anxiety and depression (from mild to severe) were 29.6% and 11.0% respectively while the prevalence of susceptibility to smoking was 16.2%. Adolescents who were exposed to SHS were significantly more likely to report depressive symptoms (p=0.050), while those who lived in cramped accommodations (p=0.023) and those who were exposed to SHS (p=0.041), were significantly more likely to report susceptibility to smoking. In multivariable analyses, indoor SHS exposure for ≥ 1 hour daily was associated with increased odds for susceptibility to smoking (AOR=3.793; 95%-CI: 0.98-14.60; p= 0.052) and increased odds for anxiety (AOR=1.303; 95%-CI: 0.84-2.01; p= 0.228) and slightly reduced odds for depression (AOR=0.952; 95%-CI: 0.62-1.47; p= 0.822) (Table 3). Table 4 displays the predictors of susceptibility to smoking cigarettes among the sampled adolescents. There was a significant association (p<0.05) between the type of accommodation (AOR: 1.40; CI: 1.037-3.079), severe depression (AOR: 2.7; CI: 1.531-5.473) having a parent that smoke (AOR: 2.76; CI: 2.506- 9.709) and having parents smoking cigarettes indoors (AOR: 4.85; CI: 3.590-21.246) with increased susceptibility to smoking among the adolescents. There were also increased odds of susceptibility to smoking among those whose mother (AOR: 1.30; CI: 0.702-3.824) and father (AOR: 2.12; CI: 0.819- 5.493) had <12 years of formal education, although, the association was not significant.

**Table 1:** association between secondhand smoke exposure and socio-demographic characteristics of respondents

	Total (group)		Secondhand smoking exposure				χ^2^	p-value
Characteristic	n	%	Daily	Often (3-6 days)	Rarely (1-2 days)	Unexposed		
**Age**	**300**	**100**	**9 (3.0)**	**23 (7.6)**	**34 (11.4)**	**234 (78.0)**		
12-14	278	92.7	9 (3.0)	23 (7.6)	27 (9.1)	219 (73.0)	0.323	0.733
≥15	22	7.7	0 (0.0)	0 (0.0)	7 (2.3)	15 (5.0)		
**Gender**								
Male	141	47.0	3 (1.0)	6 (2.7)	15 (5.0)	117 (39.0)	7.652	**0.022**
Female	159	53.0	6 (2.0)	17 (4.9)	19 (6.4)	117 (39.0)		
χ^2^; p value								
**Paternal education**								
≤12 years of formal education	110	36.7	3 (1.0)	18 (6.0)	32 (10.7)	57 (19.0)	2.951	0.249
>12 years of formal education	190	63.3	6 (2.0)	5 (1.6)	2 (0.7)	177 (59.0)		
**Maternal education**								
≤12 years of formal education	175	58.3	3 (1.0)	8 (2.7)	19 (6.4)	145 (56.2)	2.283	0.419
>12 years of formal education	125	41.7	6 (2.0)	15 (4.9)	15 (5.0)	89 (29.8)		
**Paternal occupation**								
Skilled/unskilled labour	193	64.3	8 (2.7)	14 (4.6)	10 (3.5)	161 (53.6)	3.086	0.138
Professional/ managerial	107	35.7	1 (0.3)	9 (3.0)	24 (7.9)	73 (24.4)		
**Type of accommodation**								
Room/self-contained	151	51.3	7 (2.3)	18 (6.0)	23 (7.7)	103 (34.4)	12.336	**0.020**
Flat or duplex	149	49.7	2 (0.7)	5 (1.6)	9 (3.7)	131 (43.6)		
**Exposure to Anti-tobacco messages**								
Yes	159	53.0	5 (1.7)	6 (2.7)	10 (3.5)	138 (46.2)	4.379	0.123
No	141	47.0	4 (1.3)	17 (4.9)	24 (7.9)	96 (31.9)		
**Child’s age when caregiver started smoking (n=66)**								
≤5 years	32	48.3	9 (13.6)	14 (21.1)	6 (9.0)	3 (4.6)	5.004	**0.001**
>5 years	34	51.7	0 (0.0)	9 (13.7)	19 (28.9)	6 (9.1)		
*Significant								

**Table 2:** association between anxiety, depression, susceptibility to smoking and the socio-demographic characteristics of respondents

	Total group		Generalized anxiety disorder-7		Patient health questionnaire-9		Smoking susceptibility	
Characteristic	n	%	Anxiety	No anxiety	Depression	No depression	Susceptible	Not susceptible
**Age**	**300**	**100**	**89 (29.6%)**	**211 (70.4%)**	**33 (11%)**	**267 (89%)**	**49 (16.2%)**	**251 (83.8%)**
10-14	278	92.7	87 (27.3)	191 (63.4)	21 (7.0)	260 (85.3)	46 (15.2)	232 (77.5)
≥15	22	7.7	2 (0.7)	20 (7.0)	12 (4.0)	7 (3.7)	3 (1.0)	19 (6.3)
χ^2^; p-value			9.353	0.616	13.351	0.204	5.743	0.210
**Gender**								
Male	141	47.0	53 (13.9)	89 (29.5)	14 (4.6)	127 (42.4)	30 (10.0)	111 (37.0)
Female	159	53.0	36 (15.7)	123(40.9)	19 (6.4)	140 (46.6)	19 (6.2)	140 (46.8)
χ^2^; p-value			9.302	0.729	9.473	0.602	3.497	0.467
**Paternal education**								
≤12 years of formal education	110	36.7	62 (20.6)	48 (16.1)	20 (6.8)	90 (29.9)	28 (9.2)	36.6
>12 years of formal education	190	63.3	27 (9.0)	163 (54.3)	13 (4.2)	177 (59.1)	21 (7.0)	47.3
χ^2^; p-value			20.108	0.949	29.513	0.741	8.474	0.489
**Maternal Education**								
≤12 years of formal education	175	58.3	69 (22.8)	106 (35.5)	18 (6.0)	157 (52.3)	25 (8.3)	150 (50.3)
>12 years of formal education	125	41.7	20 (6.8)	105 (34.9)	15 (5.0)	110 (36.7)	24 (7.9)	101 (33.8)
χ^2^; p-value			25.533	0.979	4.409	0.930	4.944	0.964
**Paternal occupation**								
Skilled/unskilled labour	193	64.3	70 (23.2)	123 (41.1)	21 (7.0)	172 (57.3)	27 (9.0)	166 (55.3)
Professional/managerial	107	35.7	29 (6.4)	78 (29.3)	12 (4.0)	95 (31.7)	22 (7.2)	85 (28.5)
χ^2^; p-value			6.094	0.597	3.650s	0.940	4.602	0.883
**Type of accommodation**								
Room/ self-contained	151	51.3	62 (20.8)	89 (29.5)	18 (6.0)	133 (45.3)	30 (9.9)	121 (41.4)
Flat or Duplex	149	49.7	27 (8.8)	122 (40.9)	15 (5.0)	134 (44.7)	19 (6.3)	130 (43.4)
χ^2^; p-value			19.425	0.727	22.831	0.411	17.730	0.023
**Exposure to anti-tobacco messaging**								
Yes	159	53.0	43 (14.4)	116 (38.6)	13 (4.2)	146 (48.8)	7 (2.2)	152 (50.8)
No	141	47.0	46 (15.2)	95 (31.4)	20 (6.8)	121 (40.2)	42 (14.0)	99 (33.0)
χ^2^; p-value			24.378	0.268	21.109	0.424	5.893	0.680
**Secondhand smoking exposure**								
Daily	9	3.0	9 (3.0)	0.0	9 (3.0)	0 (0.0)	9 (3.0)	0 (0.0)
Often (3-6 days)	23	7.6	20 (6.6)	3 (1.0)	14 (4.8)	9 (2.8)	(23 7.6)	0 (0.0)
Rarely (1-2 days)	34	11.4	27 (9.0)	7 (2.4)	9 (3.2)	25 (8.2)	23 (5.6)	11 (3.8)
Unexposed	234	78.0	33 (11.0)	201 (67.0)	0 (0.0)	234 (78.0)	0 (0.0)	234 (78.0)
χ^2^; p-value			16.573	0.213	14.913	0.050	10.747	0.041
**Child’s age when caregiver started smoking (n=66)**								
≤5 years	32	48.3	7 (10.7)	25 (37.6)	5 (8.3)	27 (40.0)	6 (9.0)	26 (39.3)
>5 years	34	51.7	12 (18.9)	20 (32.8)	2 (2.7)	32 (49.0)	5 (7.2)	29 (44.5)
χ^2^; p-value			12.170	0.728	16.597	0.384	4.137	0.609

**Table 3:** multivariable association between secondhand smoke exposure, anxiety, depression and susceptibility to smoking

Secondhand smoke exposure	OR	95% CI		P-value	AOR	95% CI		P-value
		Lower	Upper			Lower	Upper	
**Patient susceptibility**								
Not susceptible	1 (ref)				1 (ref)			
Susceptible	1.333	0.687	3.766	0.067	3.793	0.987	14.579	0.052
**Depression**								
No depression	1 (ref)				1 (ref)			
Depression	0.865	0.220	1.452	0.269	1.303	0.847	2.005	0.228
**Anxiety**								
No anxiety	1 (ref)				1 (ref)			
Anxiety	0.409	0.219	0.541	0.995	0.952	0.619	1.463	0.822

## Discussion

Secondhand smoke contains the same injurious chemicals inhaled by active smokers, thus those who are exposed to it encounter a comparable hazard [33,34]. Of the 300 adolescents surveyed in this study, 22% were exposed to SHS of which 11.4% were rarely exposed, 7.6% were often exposed while 3.0% of the adolescents were daily exposed to SHS indoors. This finding is comparable to the figures presented by Centers for Disease Control and Prevention (CDC) which showed that almost 50% of young adolescents are exposed to SHS at home [35]. Of the six WHO regions, the European Region (77.8%) had the highest level of exposure to SHS while the African region (27.6%) had the lowest. The prevalence of self-reported anxiety and depression (from mild to severe) among the adolescents was 29.6% and 11.0% respectively. Depression is one of the most common mental health problems, with an estimated 350 million people affected globally [36]. The findings from this study agrees with that reported by the World Health Organization which documented the prevalence of depression among adolescents around the world as between 5-10% [37]. Furthermore, the prevalence of susceptibility to smoking was 16.2%. This figure is slightly lower than that obtained from that of a US National survey of teens which obtained a prevalence of 22% for females and 23% for males [38]. In the non-smoking population, susceptibility is an initial phase in a sequence of cognitive changes, which ultimately progresses into experimentation with cigarettes, steady smoking, and eventually addiction to tobacco. Hence, any intervention that addresses this crucial first stage of susceptibility, will likely result in a reduction in the uptake of cigarettes by a significant proportion of youth [38]. In the bivariate analysis, females, those living in cramped accommodations and those who were less than 5 years of age when their caregivers started smoking indoors had significantly higher levels of exposure to SHS. The intensity of exposure to SHS and the number of individuals residing in an accommodation have a direct dose-response relationship [39]. The primary source of exposure to SHS for children living with adult smokers in cramped households is the mainstream and side stream smoke [39]. Secondhand smoke exposure was associated with slightly reduced odds for anxiety in this study but 1.3 increased odds for depression. Some authors have observed an augmented risk for anxiety with tobacco smoking [40] while quitting smoking was associated with a reduction in this risk [41]. In a study conducted by Amering *et al*., it was reported that 72% of panic disorder patients stated that they were regular smokers at the onset of their condition [42]. A previous study using US National Health and Nutrition Examination Survey data reported that SHS exposure was positively associated with major depressive disorder, generalized anxiety disorder and attention-deficit/hyperactivity disorder in adolescents [43]. Adolescents with higher frequency of SHS have also been found to be more likely to report depressive symptoms [19]. In addition, a 12-year population-based follow-up study observed that suicide mortality was higher in adolescents exposed to secondhand smoke, even with mild exposure [44].

There are numerous likely mechanisms for the association of SHS exposure with depressive symptoms even though the direction of the association and potential underlying mechanisms are not fully understood. SHS comprises electrophiles, free radicals, and metal ions that act as oxidizing or reducing agents that alter the body´s pro- anti-oxidant balance, leading to potential damage in biological molecules. Free radicals in tar such as hydroquinone and catechol can also induce oxidative stress in cells. SHS exposure also elevates the levels of adrenocorticotropic hormones which are related to mood and behavior [45-47]. Exposure to SHS in stressful states is likewise associated with an increased risk for negative lifestyle and behavioral problems such as emotional disorders at a young age [46]. Adolescent depression is an antecedent of many adverse outcomes in adulthood, and globally imposes a significant economic burden not only on individuals with the condition, but also on their families and communities [48]. Those who were exposed to SHS, were also significantly more likely to report susceptibility to smoking. Also, there was a significant association between living in a cramped accommodation, severe depression, having a parent that smokes and having parents smoking cigarettes indoors with increased susceptibility to smoking among the adolescents. In multivariable analyses, indoor SHS exposure for ≥ 1 hour daily was associated with 3.79 increased odds for susceptibility to smoking. A review of research has found that children exposed to smoking are significantly more likely to take up smoking themselves [49]. This was in agreement with a previous study that documented that children whose parents smoked were at a three-fold increased risk of smoking uptake. Children were further found to be over 70% more likely to start smoking if just one parent smoked, and over twice as likely if that parent was the mother [50].

Previous review studies have demonstrated an association between SHS exposure and smoking uptake [51]. Prior research has indicated that smoking susceptibility is a significant independent predictor of future initiation into tobacco use and that SHS could influence tobacco use and smoking behaviors [52]. Secondhand Smoke, through repeated nicotine exposure, can induce tolerance to nicotine´s adverse effects, resulting in nicotine-induced behavioral changes [53]. Close to a hundred thousand young people commence the smoking habit daily and most of them are from LMICs and pre-adolescent [54]. Adolescents who smoke are 3 times more like than nonsmokers to use alcohol, 8 times more likely to use marijuana and 22 times more likely to use cocaine. The present study suggests a positive association between SHS exposure with increased susceptibility to smoking, slightly reduced odds for anxiety but increased odds for depression. Evidence shows that smoking cessation is associated with reduction in depression, anxiety and stress and improved quality of life for people with mental health conditions and those without [55]. Cigarette smoke contains many substances that may either directly (free radicals) or indirectly (metals) exert negative effects on stress pathways, immune and mitochondrial functions, and also possibly influence neurodevelopmental and subsequent anxiety risk. Anti-tobacco messages (ATM) have primarily communicated the physical risks of smoking [56] and further cohort studies on this crucial research question may confirm the need to expand these messages to include the mental health risks of tobacco use, such as “SHS exposure is associated with increased risk of anxiety”. There are however some limitations associated with this study. Firstly, access to direct measures and biological indicators such as serum cotinine, urinary cotinine, serum carboxyhemoglobin or household air nicotine levels could have provided more valid evidence of levels of SHS exposure. Secondly, we are unable to determine the direction of the association since some parents who are smokers may have higher rates of depression, triggering indirect psychological impacts on their children who were similarly exposed to SHS. Moreover, other confounding indicators that could be associated with anxiety, depression and susceptibility to smoking such as alcohol consumption, illicit drug use, and intrapersonal factors such as stress were not determined. This baseline study however provides very important baseline data for further analytical studies.

## Conclusion

Secondhand smoke exposure in this study was associated with higher odds of susceptibility to smoking cigarettes and depression among adolescents exposed to SHS, especially among females, those living in cramped accommodations, having parents smoking cigarettes indoors and having poorly educated parents. Further validation of this findings should however be determined by cohort study designs.

### What is known about this topic

- *Secondhand smoke (SHS) exposure is one of the important risk factors that might increase susceptibility to smoking and nicotine dependence among those who have begun smoking; the importance of susceptibility to smoking within the developmental context of adolescence is significant given the role of behavioural intentions in predicting future behaviour;*
- *Previous research has shown that a disproportionate number of those aged 15 years and over who smoke (approximately 80%) live in LMICs*.

### What this study adds

- *This article scientifically presents data on the prevalence of mental health problems associated with SHS exposure;*
- *Secondhand smoke exposure in this study was associated with slightly reduced odds for anxiety in this study but 1.3-fold increased odds for depression;*
- *Those who were exposed to SHS, were also significantly more likely to report susceptibility to smoking; also there was a significant association between living in a cramped accommodation, having a parent that smokes and having parents smoking cigarettes indoors with increased susceptibility to smoking among the adolescents*.
